# The Diagnostic Value of the Random Urine Potassium‒Creatinine Ratio to the Synchronous Serum Potassium Concentration Squared for Renal Potassium Loss in Hypokalemia Patients

**DOI:** 10.1155/ije/9911400

**Published:** 2025-09-09

**Authors:** Xinyi Wang, Fei Ding, Xinyi Huang, Yong He, Guixing Li

**Affiliations:** Department of Laboratory Medicine, West China Hospital, Sichuan University, Chengdu, Sichuan, China

**Keywords:** emergency, hypokalemia, potassium‒creatinine ratio, random urine, renal potassium loss

## Abstract

**Background:** Few parameters are available for diagnosing renal potassium loss in emergency patients or patients receiving treatment. This study aimed to investigate the ratio of random urine potassium‒creatinine to the synchronous serum potassium concentration squared ([UK/UCr]/SK^2^) and compare it with other parameters in the diagnostic ability of renal potassium loss.

**Methods:** This single-center study enrolled 380 subjects, including 218 hypokalemia patients (91 with nonrenal potassium loss and 127 with renal potassium loss) and 162 normal potassium controls. The values of serum and urine were based on laboratory data. Groups were compared in pairs, and the ROC curve analysis was used to evaluate the predictive ability of parameters related to renal potassium loss.

**Results:** (UK/UCr)/SK^2^ was significantly elevated in potassium loss patients, especially in females. Moreover, a greater (UK/UCr)/SK^2^ ratio was observed in those with nonrenal potassium loss, which demonstrated a trend toward increases in the normal potassium, nonrenal potassium loss, and renal potassium loss groups among males and females. Ultimately, the AUC of (UK/UCr)/SK^2^ was the highest at 0.880 (95% CI: 0.822–0.938) in males and 0.878 (95% CI: 0.831–0.924) in females for potassium loss diagnosis.

**Conclusion:** Random (UK/UCr)/SK^2^ has good diagnostic value for renal potassium loss in patients with hypokalemia. Given that these serum and random urine parameters are easily obtainable from patients during treatment, regularly observing (UK/UCr)/SK^2^ may prove to be an effective indicator.

## 1. Introduction

Hypokalemia occurs when the potassium concentration in the serum falls below 3.5 mmol/L [1]. This topic is attracting greater attention due to the substantial evidence that there is a correlation between low potassium concentrations and adverse clinical outcomes [2–6]. Additionally, the incidence of hypokalemia is relatively high, accounting for approximately 14% of outpatients [7] and 21% of inpatients [8]. Early comprehensive analysis and rapid identification of the underlying cause are highly important for improving the prognosis of hypokalemia. However, the etiology of hypokalemia is highly complex, and determining whether a patient has renal potassium loss is a crucial initial step.

At present, 24-h urinary potassium (24H-UK) is widely used to assess renal potassium loss [9]. However, 24H-UK measurement is based on the collection and estimation of 24H-UK excretion after the patient's potassium supplementation has returned to normal, which is a time-consuming process. Nonstandard operations may lead to unnecessary diagnostic procedures. As a result, its application in outpatient or emergency patients is limited.

Spot urine samples are easy to obtain from outpatient and emergency patients. Because creatinine excretion is constant in people with a normal estimated glomerular filtration rate (eGFR) [10], the potassium‒creatinine ratio (UK/UCr) can eliminate the effects of urine concentration and dilution. Some studies have shown that the spot UK/UCr is positively correlated with 24H-UK, which is likely an alternative indicator for diagnosing potassium deficiency due to renal loss [11, 12], but more research evidence is still needed to support these findings. In addition, Elisaf and Siamopoulos reported that the fractional excretion of spot urine potassium (FEK) was markedly correlated with renal potassium loss [13]. The two indicators are used to collect samples prior to the commencement of therapy, as the influence of potassium supplementation cannot be circumvented. Interestingly, a study showed that the number of UKs per hour may serve as a marker for renal potassium loss during treatment [14]. Nevertheless, the number of UKs per hour is employed as an indicator for the evaluation of hypokalemia during the initial eight-hour period. Consequently, a straightforward and efficacious indicator for diagnosing renal potassium loss, which can be employed during treatment, has emerged as a clinical need.

In this study, we focused on a biomarker with the least impact on potassium supplementation during treatment and proposed a new ratio of the random UK/UCr to the synchronous serum potassium concentration squared ([UK/UCr]/SK^2^). In addition, we compared this ratio with relevant laboratory indices and assessed its clinical value as a diagnostic biomarker for renal potassium loss, with the goal of offering better diagnoses in this research.

## 2. Materials and Methods

### 2.1. Human Studies

**Figure 1:**
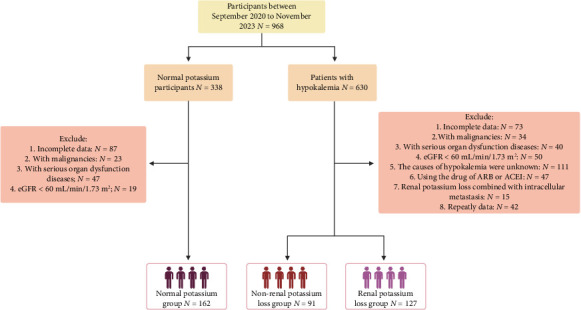
Flowchart of the study subjects screening process.

The study enrolled patients with diagnoses of hypokalemia and normal potassium at the West China Hospital of Sichuan University from September 2020 to November 2023. Serum potassium levels under 3.5 mmol/L indicated a diagnosis of hypokalemia. The controls had a normal potassium concentration (normal range: 3.5–5.5 mmol/L). All hypokalemic patients enrolled were not considered for potassium supplementation. By a series of examinations or imaging techniques, patients were categorized into two groups according to the cause of hypokalemia: those with renal potassium loss and those with extrarenal potassium loss. Etiology of renal potassium loss: the cause of the use of diuretic drugs, genetic syndromes affecting renal function, kidney diseases with renal potassium loss, and so on. Etiology of nonrenal potassium loss: decreased potassium intake, vomiting or diarrhea, excessive transcellular potassium shifting into cells, gastrointestinal loss, alkalosis, use of insulin, and so on. The etiology of all diseases should be diagnosed according to appropriate guidelines and expert consensus. The criteria for excluding patients were ① patients who were repeatedly hospitalized; ② patients without complete anthropometric parameters or basic information; ③ patients who had repeated examinations (only retained the first data); ④ patients whose eGFR was < 60 mL/min/1.73 m^2^; ⑤ patients with diabetes; ⑥ patients who used drugs that facilitate potassium excretion; ⑦ patients with malignancies or other serious organ dysfunction diseases; and ⑧ those with a body mass index (BMI, weight/height^2^) < 18.5. This research received approval from the Ethics Committee of West China Hospital, Sichuan University (2022--433). We ensured compliance with all applicable regulations in our methods.  Figure 1 displays the detailed inclusion and exclusion criteria.  Figure 1 shows the specific inclusion and exclusion processes for the participants in our study.

### 2.2. Clinical and Laboratory Data Collection

#### 2.2.1. Study Procedures

Anthropometric data related to individual study subjects, comprising sex, height, weight, and BMI, were collected. Moreover, 24-h urine, random urine, and blood samples were gathered. The initial morning urine sample was discarded for 24H-UK collection. In addition, random urine samples and blood samples were collected synchronously. Next, the blood was centrifuged at 3000 rpm for 10 min to obtain the serum. The urine was centrifuged at 3500 rpm for 10 min to obtain the supernatant. The serum concentrations of potassium (K), creatinine (CREA), urine biochemically measured urine K, and urine CREA were measured. Serum and urine biochemical parameters were both measured via a ROCHE 702 analyzer (Germany).

#### 2.2.2. Define

24H-UK is calculated as the product of 24-h urine output and random urine potassium (UK). UK/UCr is calculated as the ratio of the random UK concentration divided by the random urine creatinine concentration. (UK/UCr)/SK^2^ is calculated as the ratio of random UK/UCr divided by the synchronous serum potassium concentration squared. FEK refers to the fraction of potassium excreted in urine from a random sample. FEK = (UK × SCr) × 100%/(SK × UCr). BMI was determined by dividing weight by the square of height.

### 2.3. Statistical Analysis

Normally distributed continuous variables are indicated by means ± standard deviations, non-normally distributed ones by medians and interquartile ranges, and categorical variables by percentages. Continuous variables were analyzed using the Mann–Whitney *U* test or *t*-test, while categorical variables were assessed with the chi-square test. Differences between the random (UK/UCr)/SK^2^ and other indices were calculated via the Bland‒Altman plot. The relationships between (UK/UCr)/SK^2^ and other parameters were analyzed via Spearman's correlation analysis. The diagnostic performance of the parameter was evaluated using ROC curves. Statistical analysis was performed via R software (Version 3.6.0) and GraphPad Prism software (Version 8.0). All the statistical tests are bilateral probability tests, with a *p* value of < 0.05 indicating statistical significance.

## 3. Results

### 3.1. Characteristics of the Enrolled Patients

The characteristics of 218 hypokalemic patients (91 with nonrenal potassium loss and 127 with renal potassium loss) and 162 normal potassium controls are summarized in Table 1. In the cohort, the primary etiology of renal potassium loss was primary hyperaldosteronism (PA), which was identified in 66.1% (*n* = 84) of the patients. Other etiologies included renal tubular acidosis (RTA), Gitelman syndrome, Cushing syndrome, primary adrenal hyperplasia, the use of diuretics, and Bartter syndrome (*n* = 7, 6, 9, 12, and 7, respectively). The etiologies of the nonrenal potassium loss group included hyperinsulinemia (*n* = 25), diabetes (*n* = 20), thyrotoxic periodic paralysis (*n* = 15), hypokalemic periodic paralysis (*n* = 18), vomiting or diarrhea (*n* = 7), and alkalosis (*n* = 6). Among all the subjects in this study, 43.9% (167/380) were male, with an average age of 45 years and ranging from 14 to 85 years. The three groups did not show any statistically significant differences in age, gender distribution, or BMI. With respect to laboratory parameters, higher levels of UK/UCr, (UK/UCr)/SK2, FEK, and 24H-UK were found in the renal potassium loss group, but they had lower SK, UK, and UCr levels than the other groups (*p* < 0.01). Surprisingly, (UK/UCr)/SK^2^ in nonrenal potassium loss patients was significantly greater than that in normal potassium individuals; however, there were no significant differences in the renal function-related indicators SCr and eGFR between the different groups.

### 3.2. Renal Potassium Loss-Related Parameter Levels in the Enrolled Patients

**Figure 2:**
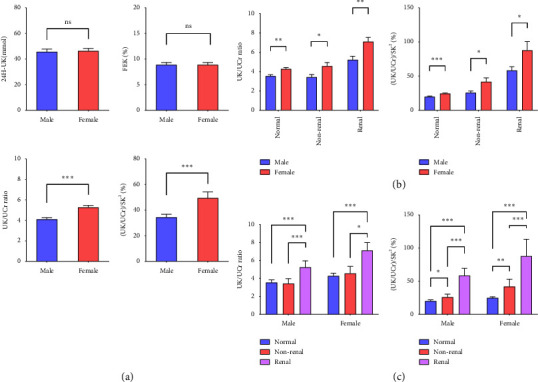
The distribution of renal potassium loss parameters in enrolled subjects. (a) 24H-UK, FEK, UK/UCr, and (UK/UCr)/SK^2^ distribution in male and female subjects. (b) UK/UCr and (UK/UCr)/SK^2^ distribution in male and female subjects of the normal potassium group, nonrenal potassium loss group, and renal potassium loss group. (c) UK/UCr and (UK/UCr)/SK^2^ distribution in the normal potassium group, nonrenal potassium loss group, and renal potassium loss group for male and female, respectively. ns: *p* > 0.05; ∗*p* < 0.05; ∗∗*p* < 0.01, and ∗∗∗*p* < 0.001.

We initially investigated the levels of four parameters among sexes. As shown in Figure 2(a), there was a significant increase in UK/UCr and (UK/UCr)/SK^2^ levels in females compared with those in males (*p* < 0.05), and no major differences were observed in FEK or 24H-UK (*p* > 0.05). Further analysis revealed that UK/UCr and (UK/UCr)/SK^2^ were also greater in females than in males in the three groups. The distributions of the two parameters among the sexes in the three groups are illustrated in Figure 2(b). These two parameters were significantly elevated in both males and females in the renal potassium loss group. Moreover, (UK/UCr)/SK2 was the only parameter elevated in the nonrenal potassium loss group compared with the normal potassium group, which demonstrated a trend toward an increase in the normal potassium, nonrenal potassium loss, and renal potassium loss groups among males and females (*p* < 0.05) (Figure 2(c)).

### 3.3. Evaluation of the Diagnostic Efficacy of Laboratory Parameters for Renal Potassium Loss

**Figure 3:**
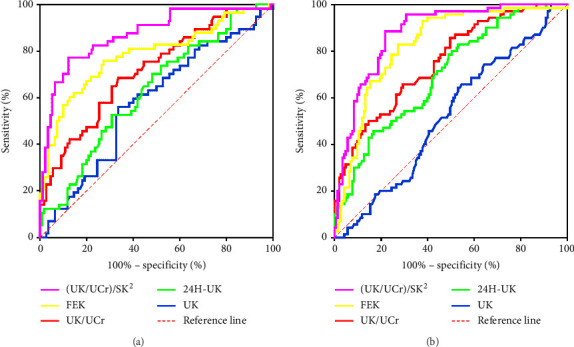
ROC curves of five parameters for the diagnosis of renal potassium loss in hypokalemia. (a) Males. (b) Females.

Table 2 provides a summary of the ROC analysis results for different parameters used to diagnose renal potassium loss in hypokalemic male and female patients.  Figure 3 displays the ROC curve. The results revealed that (UK/UCr)/SK^2^ had better diagnostic efficacy than the other parameters for renal potassium loss. The AUC of (UK/UCr)/SK^2^ was the highest at 0.880 (95% CI: 0.822–0.938) in males and 0.878 (95% CI: 0.831–0.924) in females. Youden's index, calculated by adding sensitivity and specificity and then subtracting one, was employed to determine the cutoff value. The cutoff value of (UK/UCr)/SK^2^ was 27.02%, with a sensitivity of 80.70% and a specificity of 80.91% for males, and 35.58%, with a sensitivity of 88.57% and a specificity of 78.32% for females, respectively.

### 3.4. Correlations of (UK/UCr)/SK^2^ With Relevant Parameters

**Figure 4:**
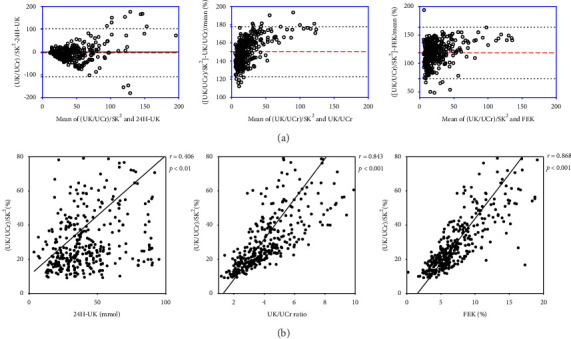
The agreement and correlation of relevant parameters in potassium renal loss. (a) Bland–Altman plot of comparison of random (UK/UCr)/SK^2^ with 24H-UK, UK/UCr, and FEK ratio, respectively. (b) Correlation curves of random (UK/UCr)/SK^2^ with 24H-UK, UK/UCr ratio and FEK ratio, respectively.

Bland‒Altman analysis revealed that these parameters were in good agreement with each other. The findings indicate that only 4.21% (16/380) of the patients exhibited a difference greater than ±2 SDs from the mean between the (UK/UCr)/SK^2^ and UK/UCr, and 3.42% (13/380) of the patients presented a difference greater than ± 2 SDs from the mean between the (UK/UCr)/SK^2^ and 24H-UK ratios and 3.68% (14/380) between the (UK/UCr)/SK^2^ and random FEK ratios (Figure 4(a)). The results of Spearman's correlation analysis indicated that the (UK/UCr)/SK^2^ ratio exhibited a positive correlation with the 24H-UK ratio (*r* = 0.406, *p* < 0.01), the UK/UCr (*r* = 0.843, *p* < 0.001), and the FEK ratio (*r* = 0.868, *p* < 0.001) (Figure 4(b)).

## 4. Discussion

The most prevalent electrolyte disorder encountered in clinical settings is hypokalemia, possibly leading to adverse events for multiple systems [15–17]. Overall, this study aimed to identify a rapid and convenient biomarker for diagnosing potassium loss. In the present study, the (UK/UCr)/SK^2^ ratio was notably higher in the group with renal potassium loss compared with the groups with nonrenal potassium loss and normal potassium levels. Similarly, there were consistent observations in men and women. Moreover, we compared the changes in various laboratory parameters associated with renal potassium loss in the three groups and further elucidated the diagnostic value of (UK/UCr)/SK^2^. Notably, our results show that the diagnostic efficacy of random (UK/UCr)/SK^2^ was superior to that of 24H-UK, random UK/UCr, and random FEK.

In total, a relatively large cohort was enrolled over a three-year period, including 127 renal potassium loss patients, 162 normal potassium controls, and 91 nonrenal potassium loss controls. The prevalence of hypokalemia was greater in females (55.5%) than in males (45.5%), which was consistent with the findings reported by Hawkins [18]. Given the difference in creatinine excretion between men and women [19], we undertook a comparative analysis of (UK/UCr)/SK^2^ across the three sex-based groups. The results revealed that random (UK/UCr)/SK^2^ is particularly sensitive for diagnosing renal potassium loss, with values of 80.70% in males and 88.57% in females. Regulation of potassium levels is a primary role of the kidney, and excessive urinary loss of potassium may result in hypokalemia [20]. Increased excretion occurs in PA patients due to renal potassium loss [21], and the elevation in serum potassium levels subsequent to potassium supplementation is along with a rise in excretion and the concentration of UK/UCr. Owing to the physiology of potassium homeostasis [22], the (UK/UCr)/SK^2^ ratio should also be increased. This hypothesis was supported by our results. Because diuretic drugs [22], Gitelman syndrome [9], and RTA [23] are used with renal potassium loss, the UK/UCr/SK^2^ ratio is significantly elevated. In the group experiencing nonrenal potassium loss, the (UK/UCr)/SK^2^ ratio was elevated compared with normal potassium levels and control group. For hyperinsulinaemia patients, the primary function of insulin is to facilitate the uptake of potassium from the inside to the outside [24], and severe intracellular transfer of potassium can lead to hypokalemia. This phenomenon is also observed in patients with thyrotoxic periodic paralysis [25] and hyperglycemia [26]. Although there was no difference in the UK/UCr between the two groups, the aforementioned factors may account for the observed increase in the median (UK/UCr)/SK^2^ ratio in the nonrenal potassium loss group. To our knowledge, this is the first ratio parameter used to distinguish nonrenal potassium loss from normal potassium.

The indicator of random (UK/UCr)/SK^2^ was initially proposed in our study. Traditionally, the cutoff value of 24H-UK was 15 or 20 mmol/d [1, 27] in clinical practice. We found that the cutoff was above 35.43 mmol per day in males and 35.65 mmol per day in females, which is similar to the discoveries of several studies [28, 29]. The elevated 24H-UK may be due to the immediate replacement of potassium during 24-h urine collection. Our hypothesis aligns with that of Gumz et al., who reported that the excretion of potassium may be > 20 mmol with rapid and substantial replacement of potassium [30], which may lead to a misjudgment of renal potassium loss. In addition, several random urine parameters were included in our study. Potassium loss from nonrenal origins is indicated by a random urine potassium concentration of less than 20 mmol/L [31]. In our investigation, the UK concentration was significantly elevated in the normal potassium group compared with the other two groups, but the random UK concentration did not significantly differ between those with nonrenal potassium loss and those with renal potassium loss. Patients with chronic hypokalemia may present with polyuria due to thirst, resulting in low urine potassium concentrations despite severe renal potassium losses [32], and potassium secretion and excretion exhibit a significant circadian rhythm and dietary vulnerability [33], which could provide an explanation for the observed results. In contrast, 24H-UK is more accurate than random UK levels.

In the past decade, some researchers have reported that spot UK/UCr, random urine prior to treatment, may be a new indicator of renal potassium loss. A UK/UCr threshold of 2.5 successfully identified patients experiencing potassium loss by Lin et al. [34] and 1.5 by Assadi [35], which is different from our results of 3.78 in males and 4.89 in females. We speculate that the discrepancies might be due to population, age, and urine collection time. Although this indicator is more convenient than 24-h urine collection is, it is not suitable for critically ill patients requiring immediate potassium supplementation or for patients requiring continuous potassium supplementation of unknown etiology. Furthermore, various formulas have been created and confirmed for estimating 24-h urinary potassium excretion using random urine samples [36–38]. However, the Bland‒Altman plot revealed a considerable discrepancy between the predicted value derived from the formula and the 24H-UK of the observed excretions in the Hooft van Huysduynen et al.'s study [11]. In our study, only 3.42% (13/380) of the patients exhibited a difference between the (UK/UCr)/SK^2^ and 24H-UK.

We noted that the random FEK ratio used to diagnose potassium loss was 7.49%, with a sensitivity of 74.14% and a specificity of 73.64% for males, and the cutoff value was 7.49%, with a sensitivity of 82.86% and a specificity of 71.33% for females, which was second only to random (UK/UCr)/SK^2^. In contrast to earlier findings, however, the spot ratio at a threshold of 9.29% with a sensitivity and specificity of 80.60% and 85.70% [29] was used in the Li et al.'s study, and when a cutoff of 9.80% was used in the Yuan et al.'s study, sensitivity was measured at 86.70% while specificity was 87.10% [28]. These findings indicate that the collection of samples may influence the diagnostic efficacy of FEK, with spot urine demonstrating superior performance to random urine. In instances where patients are receiving potassium supplementation, the diagnostic efficacy of FEK is diminished, with random (UK/UCr)/SK^2^ emerging as a more reliable indicator.

Overall, we found that the random (UK/UCr)/SK^2^ ratio was the most effective for identifying patients experiencing renal loss of potassium in hypokalemia, followed by the random FEK ratio, the UK/UCr ratio, the 24H-UK ratio, and the UK. The following three characteristics render random (UK/UCr)/SK^2^ as a potentially valuable diagnostic indicator for renal loss patients: (1) collection of random urine and serum is convenient in outpatient and emergency patients. (2) Some patients who require continuous potassium supplementation are not affected. (3) The only parameter to demonstrate a trend toward an increase in the normal potassium, nonrenal potassium loss, and renal potassium loss groups.

### 4.1. Limitations

We acknowledge several limitations in our study. The foremost is the limited patient enrollment, resulting from the extended time required for diagnosing the specific etiology, which may introduce selection bias. Second, we did not subgroup the hypokalemic patients according to severity. Finally, there is a need for larger samples and multicenter studies to further confirm the findings of this study.

## Figures and Tables

**Table 1:** Comparison of clinical, laboratory characteristics between patients with hypokalemia and normal potassium controls.

Characteristics	Normal potassium group	Nonrenal potassium loss group	Renal potassium loss group
	*n* = 162	*n* = 91	*n* = 127
Male (%)	43.2	45.1	44.9
Age (y)	45.50 (35.25, 55.00)	45.00 (34.50, 54.00)	47.00 (34.00, 54.00)
BMI (kg/m^2^)	22.92 (21.27, 25.13)	23.30 (21.05, 26.35)	23.28 (21.26, 25.90)
SK (mmol/L)	4.19 (3.98, 4.40)	3.63 (3.30, 4.07)	3.21 (2.90, 3.53)^∗∗##^
SCr (mmol/L)	0.07 (0.05, 0.08)	0.07 (0.06, 0.08)	0.06 (0.06, 0.08)
eGFR (mL/[min·1.73 m^2^])	106.60 (94.75, 115.55)	109.25 (98.08, 120.06)	109.52 (91.72, 117.50)
UK (mmol/L)	48.25 (33.65, 68.03)	42.33 (25.65, 53.50)^∗∗^	40.00 (29.59, 55.30)^∗^
UCr (mmol/L)	12.73 (8.85, 18.59)	11.03 (5.78, 16.81)^∗∗^	6.60 (4.45, 11.45)^∗∗##^
UK/UCr ratio	3.66 (2.94, 4.77)	3.34 (2.27, 5.09)	5.16 (3.88, 7.91)^∗∗##^
(UK/UCr)/SK^2^ (%)	20.67 (16.72, 26.78)	24.01 (17.31, 40.68)^∗^	52.82 (37.38, 79.15)^∗∗##^
FEK (%)	5.71 (4.76, 7.44)	6.07 (4.34, 10.08)	11.55 (7.89, 16.92)^∗∗##^
24H-UK (mmol)	35.35 (23.85, 51.35)	34.31 (25.95, 49.68)	46.80 (35.22, 71.42)^∗∗##^

*Note:* Compared with normal, ^∗^*p* < 0.05 and ^∗∗^*p* < 0.01. Compared with extrarenal potassium loss, ^#^*p* < 0.05 and ^##^*p* < 0.01.

**Table 2:** Outcomes of ROC analysis for different indicators in the diagnosis of renal potassium loss.

Parameters	AUC (95% CI)	Cutoff	Sens	Spec	PLR	NLR	YI
Male (*n* = 167)							
UK (mmol)	0.581 (0.490, 0.672)	42.3	56.14	66.36	1.67	0.66	22.50
UK/UCr	0.715 (0.633, 0.797)	3.78	68.42	66.36	2.03	0.48	34.78
**(UK/UCr)/**SK^2^**(%)**	**0.880 (0.822, 0.938)**	**27.02**	**80.70**	**80.91**	**4.23**	**0.24**	**61.61**
FEK (%)	0.783 (0.705, 0.862)	7.49	74.14	73.64	2.81	0.35	47.78
24H-UK (mmol)	0.628 (0.541, 0.716)	35.43	70.18	51.82	1.46	0.58	22.00
Female (n = 213)							
UK (mmol)	0.519 (0.439, 0.600)	44.25	65.71	45.45	1.20	0.75	11.16
UK/UCr	0.753 (0.686, 0.821)	4.89	65.71	70.63	2.24	0.49	36.34
**(UK/UCr)/**SK^2^**(%)**	**0.878 (0.831, 0.924)**	**35.58**	**88.57**	**78.32**	**4.09**	**0.15**	**66.89**
FEK (%)	0.827 (0.770, 0.884)	7.49	82.86	71.33	2.89	0.24	54.19
24H-UK (mmol)	0.698 (0.625, 0.770)	35.65	72.86	52.45	1.53	0.52	25.31

*Note:* Sens, sensitivity; Spec, specificity. The bold font is intended to emphasize that this indicator has the best diagnostic performance in both male and female.

Abbreviations: AUC = area under the curve, CI = confidential interval, NLR = negative likelihood ratio, PLR = positive likelihood ratio, YI = Youden's index.

## Data Availability

The corresponding author will provide the raw data supporting the conclusions of this study upon reasonable request, subject to privacy or ethical restrictions.

## References

[1] Gennari F. J. (1998). Hypokalemia. *New England Journal of Medicine*.

[2] Núñez J., Bayés-Genís A., Zannad F. (2018). Long-Term Potassium Monitoring and Dynamics in Heart Failure and Risk of Mortality. *Circulation*.

[3] Palaka E., Grandy S., Darlington O., McEwan P., van Doornewaard A. (2020). Associations Between Serum Potassium and Adverse Clinical Outcomes: A Systematic Literature Review. *International Journal of Clinical Practice*.

[4] Kovesdy C. P., Matsushita K., Sang Y. (2018). Serum Potassium and Adverse Outcomes Across the Range of Kidney Function: A CKD Prognosis Consortium Meta-Analysis. *European Heart Journal*.

[5] Usman A., Mustafa N., Iqbal S. P. (2021). Mapping the Role of pH-Adjusted Potassium in Diabetic Ketoacidosis: Hypokalemia and the Patient Outcomes. *International Journal of Clinical Practice*.

[6] Wu H., Huang R., Fan J., Luo N., Yang X. (2022). Low Potassium Disrupt Intestinal Barrier and Result in Bacterial Translocation. *Journal of Translational Medicine*.

[7] Dhondup T., Qian Q. (2017). Acid-Base and Electrolyte Disorders in Patients With and Without Chronic Kidney Disease: An Update. *Kidney Disease*.

[8] Tchounwou P., Udensi U. (2017). Potassium Homeostasis, Oxidative Stress, and Human Disease. *International Journal of Clinical and Experimental Pathology*.

[9] Molin C. Z. D., Trevisol D. J. (2017). Persistent Severe Hypokalemia: Gitelman Syndrome and Differential Diagnosis. *Jornal Brasileiro de Nefrologia*.

[10] Lin S. H., Lin Y. F., Halperin M. L. (2001). Hypokalaemia and Paralysis. *QJM: An International Journal of Medicine*.

[11] Hooft van Huysduynen E. J., Hulshof P. J., van Lee L. (2014). Evaluation of Using Spot Urine to Replace 24H Urine Sodium and Potassium Excretions. *Public Health Nutrition*.

[12] Jędrusik P., Symonides B., Wojciechowska E., Gryglas A., Gaciong Z. (2017). Diagnostic Value of Potassium Level in a Spot Urine Sample as an Index of 24-hour Urinary Potassium Excretion in Unselected Patients Hospitalized in a Hypertension Unit. *PLoS One*.

[13] Elisaf M., Siamopoulos K. C. (1995). Fractional Excretion of Potassium in Normal Subjects and in Patients With Hypokalaemia. *Postgraduate Medical Journal*.

[14] Phakdeekitcharoen B., Kreepala C., Boongird S. (2011). Urine Potassium per Hour as a Marker for Renal Potassium Losses. *Journal of the Medical Association of Thailand*.

[15] Brookes E. M., Snider J., Hart G. K., Robbins R., Power D. A. (2021). Serum Potassium Abnormalities in Chronic Kidney Disease: Prevalence, Patient Characteristics and Clinical Outcomes. *Internal Medicine Journal*.

[16] Krogager M. L., Søgaard P., Torp-Pedersen C. (2020). Impact of Plasma Potassium Normalization on Short-Term Mortality in Patients With Hypertension and Hypokalemia or Low Normal Potassium. *BMC Cardiovascular Disorders*.

[17] Hripcsak G., Suchard M. A., Shea S. (2020). Comparison of Cardiovascular and Safety Outcomes of Chlorthalidone vs Hydrochlorothiazide to Treat Hypertension. *JAMA Internal Medicine*.

[18] Hawkins R. C. (2003). Gender and Age as Risk Factors for Hypokalemia and Hyperkalemia in a Multiethnic Asian Population. *Clinica Chimica Acta*.

[19] Kestenbaum B., Ix J. H., Gansevoort R. (2022). Population-Based Limits of Urine Creatinine Excretion. *Kidney International Reports*.

[20] Gumz M. L., Lynch I. J., Greenlee M. M., Cain B. D., Wingo C. S. (2010). The Renal H+-K+-ATPases: Physiology, Regulation, and Structure. *American Journal of Physiology-Renal Physiology*.

[21] Han R., Jiang X. (2022). Hypokalemia-Induced Rhabdomyolysis as the First Symptom of Primary Aldosteronism: A Case Report and Literature Review. *Annals of Palliative Medicine*.

[22] Kardalas E., Paschou S. A., Anagnostis P., Muscogiuri G., Siasos G., Vryonidou A. (2018). Hypokalemia: A Clinical Update. *Endocrine Connections*.

[23] Palmer B. F., Kelepouris E., Clegg D. J. (2021). Renal Tubular Acidosis and Management Strategies: A Narrative Review. *Advances in Therapy*.

[24] Nguyen T. Q., Maalouf N. M., Sakhaee K., Moe O. W. (2011). Comparison of Insulin Action on Glucose Versus Potassium Uptake in Humans. *Clinical Journal of the American Society of Nephrology*.

[25] Fralick M., Sarma S. (2021). Thyrotoxic Periodic Paralysis. *New England Journal of Medicine*.

[26] Palmer B. F. (2015). Regulation of Potassium Homeostasis. *Clinical Journal of the American Society of Nephrology*.

[27] Funder J. W., Carey R. M., Mantero F. (2016). The Management of Primary Aldosteronism: Case Detection, Diagnosis, and Treatment: An Endocrine Society Clinical Practice Guideline. *Journal of Clinical Endocrinology and Metabolism*.

[28] Yuan H., Wang B., Yang Y., Li X. (2023). Diagnostic Value of the Fractional Excretion of Urine Potassium for Primary Aldosteronism. *Annals of Translational Medicine*.

[29] Li J., Ma H., Lei Y., Wan Q. (2020). Diagnostic Value of Parameters From a Spot Urine Sample for Renal Potassium Loss in Hypokalemia. *Clinica Chimica Acta*.

[30] Gumz M. L., Rabinowitz L., Wingo C. S. (2015). An Integrated View of Potassium Homeostasis. *New England Journal of Medicine*.

[31] Lim S. (2007). Approach to Hypokalemia. *Acta Med Indones*.

[32] Berl T., Linas S. L., Aisenbrey G. A., Anderson R. J. (1977). On the Mechanism of Polyuria in Potassium Depletion. *Journal of Clinical Investigation*.

[33] Zuber A. M., Centeno G., Pradervand S. (2009). Molecular Clock is Involved in Predictive Circadian Adjustment of Renal Function. *Proceedings of the National Academy of Sciences of the United States of America*.

[34] Lin S. H., Lin Y. F., Chen D. T., Chu P., Hsu C. W., Halperin M. L. (2004). Laboratory Tests to Determine the Cause of Hypokalemia and Paralysis. *Archives of Internal Medicine*.

[35] Assadi F. (2008). Diagnosis of Hypokalemia: A Problem-Solving Approach to Clinical Cases. *Iranian Journal of Kidney Diseases*.

[36] Tanaka T., Okamura T., Miura K. (2002). A Simple Method to Estimate Populational 24-h Urinary Sodium and Potassium Excretion Using a Casual Urine Specimen. *Journal of Human Hypertension*.

[37] Jędrusik P., Symonides B., Gaciong Z. (2018). Comparison of Three Formulas to Estimate 24-hour Urinary Sodium and Potassium Excretion in Patients Hospitalized in a Hypertension Unit. *Journal of the American Society of Hypertension*.

[38] Jędrusik P., Symonides B., Gaciong Z. (2019). Estimation of 24-hour Urinary Sodium, Potassium, and Creatinine Excretion in Patients With Hypertension: Can Spot Urine Measurements Replace 24-hour Urine Collection?. *Polish Archives of Internal Medicine*.

